# Validation of a prognostic model for adverse perinatal health outcomes

**DOI:** 10.1038/s41598-020-68101-3

**Published:** 2020-07-09

**Authors:** Jacqueline Lagendijk, Ewout W. Steyerberg, Leonie A. Daalderop, Jasper V. Been, Eric A. P. Steegers, Anke G. Posthumus

**Affiliations:** 1 Department of Obstetrics and Gynaecology, Erasmus MC, University Medical Centre Rotterdam, PO Box 2040, 3000 CA Rotterdam, The Netherlands; 2 Department of Public Health, Erasmus MC, University Medical Centre Rotterdam, PO Box 2040, 3000 CA Rotterdam, The Netherlands; 3 Department of Biomedical Data Sciences, Leiden University Medical Centre, PO Box 9600, 2300 RC Leiden, The Netherlands; 4 Division of Neonatology, Department of Paediatrics, Erasmus MC-Sophia Children’s Hospital, University Medical Centre Rotterdam, PO Box 2040, 3000 CA Rotterdam, The Netherlands

**Keywords:** Risk factors, Statistics

## Abstract

There is a strong association between social deprivation and adverse perinatal health outcomes, but related risk factors receive little attention in current antenatal risk selection. To increase awareness of healthcare professionals for these risk factors, a model for antenatal risk surveillance and care was developed in The Netherlands, called the ‘Rotterdam Reproductive Risk Reduction’ (R4U) scorecard. The aim of this study was to validate the R4U-scorecard. This study was conducted using external, prospective data from thirty-two midwifery practices, and fifteen hospitals in The Netherlands. The main outcome measures were the discrimination of the prognostic models for the probability of a pregnant woman developing adverse pregnancy outcomes (babies born preterm or small for gestational age), and calibration. We performed cross-validation and updated the model using statistical re-estimation of all predictors. 1752 participants were included, of whom 282 (16%) had one of the predefined adverse outcomes. The discriminative value of the original scoring system was poor [area under the curve (AUC) of 0.58 (95% CI 0.53–0.64)]. The model showed moderate calibration. The updated R4U-scorecard showed good generalisability to the validation set but did not alter the predictive value [AUC 0.61 (95% CI 0.56–0.66)]. By using external data and by updating the prognostic model, we have provided a comprehensive evaluation of the R4U-scorecard. Further improvement in classification of high-risk pregnancies is important considering the necessity of early risk detection for healthcare professionals to take appropriate actions to prevent these risks from becoming manifest problems.

## Introduction

There is a strong association between social deprivation and adverse perinatal health outcomes. This association is already present during pregnancy and extends into adulthood, with potentially severe long-term health consequences^1–5^. In The Netherlands, risk surveillance in antenatal health care traditionally mainly focuses on single medical or obstetric risk factors^6^. Psychosocial (non-medical) risk factors generally receive little attention. To increase awareness among health care professionals for these risk factors, a model for antenatal risk surveillance and care was developed in 2008 in The Netherlands^7^. This model, implemented as the ‘Rotterdam Reproductive Risk Reduction (R4U)’ scorecard (supplementary Fig. 1), estimates the probability that a pregnant woman is at increased risk of adverse pregnancy outcomes based on multiple medical, obstetric, and non-medical factors (i.e. risk factors related to a person’s socioeconomic status and environment). Additionally, the R4U-scorecard is accompanied by recommended decisions for clinicians, such as prioritisation of risk factors, risk-specific care pathways, and multidisciplinary consultations^8^.

Following its development, the R4U-scorecard was used in the national Healthy Pregnancy 4 All-1 (HP4All-1) programme, a Cluster Randomized Controlled Trial (C-RCT). This trial investigated the effectiveness of systematic risk detection and preventive strategies to reduce adverse perinatal health outcomes in antenatal healthcare^8–10^. The implementation of the R4U-scorecard into routine care, along with risk-guided care throughout pregnancy, was feasible. Moreover it had a positive impact on physicians’ behaviour by improving awareness of one of the most common adverse perinatal health outcomes during pregnancy, namely intra-uterine growth restriction^7^.

We aimed to conduct a comprehensive evaluation of the R4U. We hereto included cross-validation of the prognostic model underlying the scorecard and suggest directions for improvement by updating the model^11,12^.

## Results

Of the 2,269 women who originally participated in the intervention arm of the C-RCT embedded in the HP4All-1 programme^7^, 1752 women (77%) were included in this study. The other participants were excluded because, despite being in the intervention arm, they did not undergo antenatal risk surveillance with the R4U-scorecard. Among the included pregnancies, 282 (16%) had one of the predefined adverse perinatal health outcomes (i.e. baby born preterm or small for gestational age (SGA)). Women with an adverse outcome were more often smokers, single mothers, and more often had a net household income below 1,000 euros per month (Table 1).

**Table 1:** Patient characteristics, comparing women with and without an adverse pregnancy outcome.

	Women with adverse pregnancy outcomes (n = 282)		Women without adverse pregnancy outcomes (n = 1,470)		p value^A^
	N	%	N	%	
**Maternal characteristics**					
Age category (years)					
< 20	0	0	13	0.9	0.267
20–35	206	73.0	1,079	73.4	
> 35	76	27.0	378	25.7	
Ethnic origin					
Western	243	86.2	1,301	88.5	0.089
Non-western	39	13.8	156	10.6	
Missing	0	0.0	13	0.9	
Smoking during pregnancy					
Yes	70	24.8	248	16.9	0.005
No	210	74.5	1,202	81.8	
Missing	2	0.7	20	1.4	
Single mother					
Yes	32	11.3	76	5.2	0.001
No	250	88.7	1,392	94.7	
Missing	0	0.0	2	0.1	
Low household income					
Yes	36	12.8	113	7.7	0.013
No	245	86.9	1,343	91.4	
Missing	1	0.4	14	1.0	
BMI at start pregnancy					
BMI < 25	22	7.8	67	4.6	0.073
BMI 25–35	195	69.1	1,040	70.7	
BMI > 35	65	23.0	363	24.7	
**Pregnancy characteristics**					
Parity					
Nulliparous	128	45.4	672	45.7	0.920
Multiparous	154	54.6	798	54.3	

*SGA* small for gestational age.

^A^P-value based on chi-square analysis for categorical variables.

^B^Western versus non-western origin based on maternal country of birth and classified according to Statistics Netherlands.

^C^Low net income defined as a household income below 1,000 euro's/month.

The median R4U-score was 6 (IQR 4–9). An R4U-score above 16 points (n = 90), was associated with substantially higher odds of having an adverse pregnancy outcome [OR 3.2 (95% CI 2.1–4.8)]. In the development set for the cross validation, the median R4U score was the same as observed in the complete dataset. A high score (above 16 points) resulted in a higher odds of having an adverse pregnancy outcome in the development set [OR 4.2 (95% CI 2.1–8.1)].

**Figure 1:**
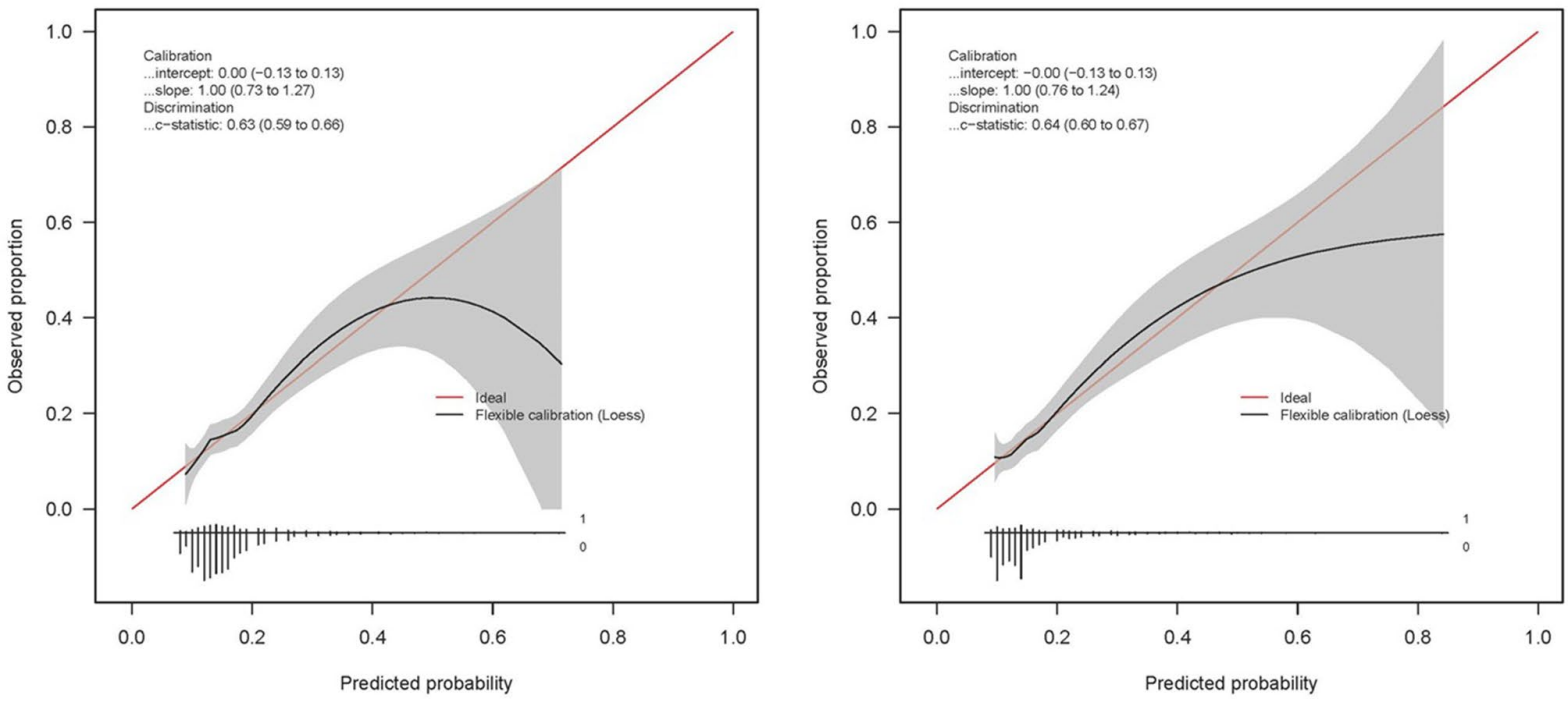
Calibration plot of the original model and the updated model. Calibration curve comparison between the original and the updated model for neonatal morbidities with 95% confidence interval in grey. The y-axis represents the observed proportion of high-risk scores (above 16 points). The intercept and slope of the logistic regression model are presented together with the c-statistic, indicating the discriminative ability. The diagonal red 45-degree line represents perfect prediction by an ideal model. The distribution of participants is indicated with spikes at the bottom of the graph, stratified by endpoint (those with neonatal morbidities above the x-axis and those without adverse outcomes below the x-axis). Graph: xlim = c(0,.45).

The original scoring system had an AUC of 0.58 (95% CI 0.53–0.64) in the validation set. The model showed moderate calibration as evidenced by the calibration plot (Fig. 1).

### Update of the original model in the development set

**Figure 2:**
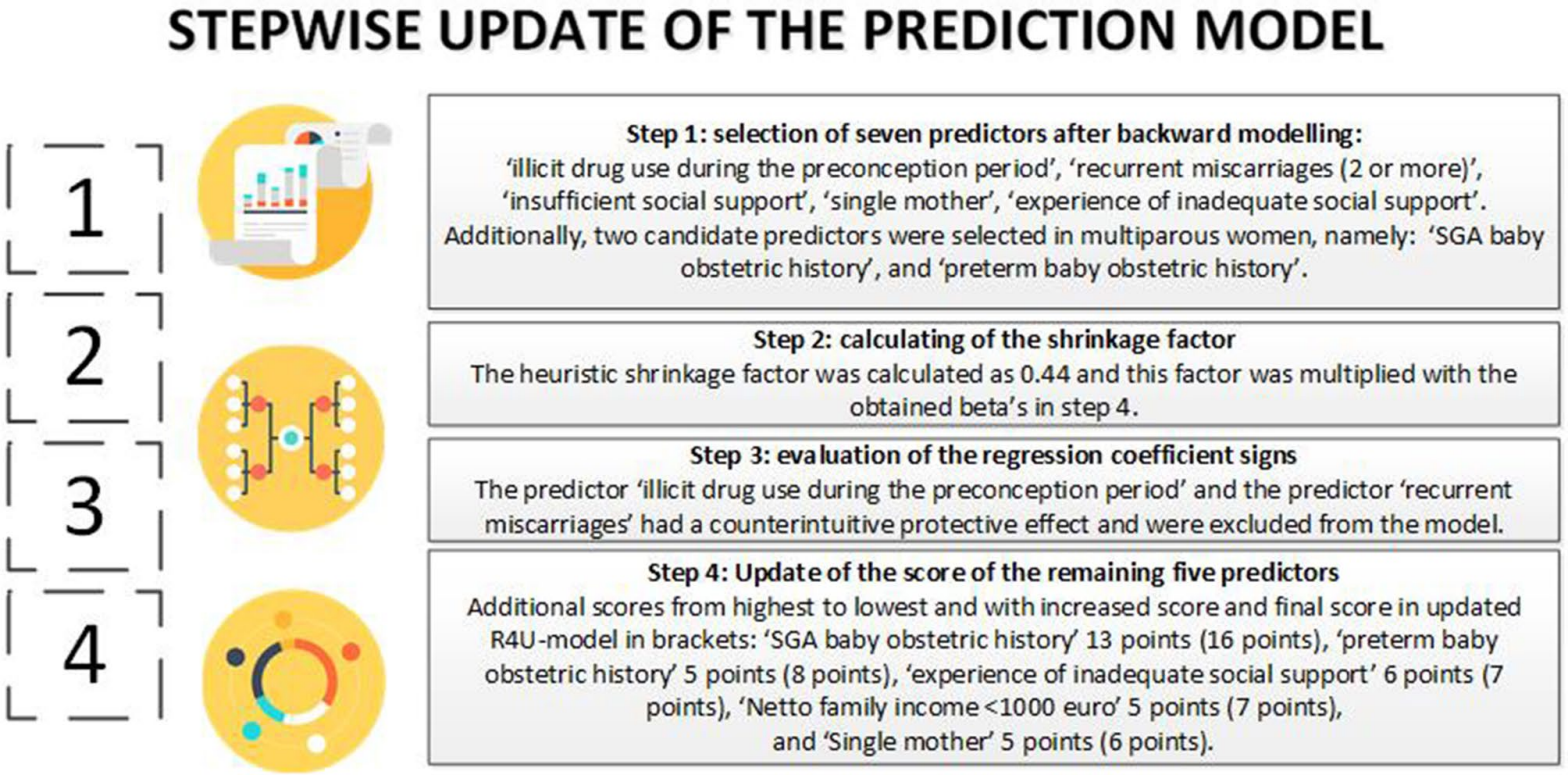
Updating steps of the prognostic model.

We selected seven predictors for which the R4U score would be updated (Fig. 2). The heuristic shrinkage factor was calculated as 0.45 (assuming 43 degrees of freedom). One point increase in R4U-score corresponded with a β-coefficient of 0.06.

Two of the seven predictors, i.e. ‘illicit drug use during the preconception period’ and ‘recurrent miscarriages’, had a counterintuitive sign (i.e. a protective effect) and were therefore excluded from the model (Fig. 2).

### Predictive value of the updated model in the validation set

Updating of the prognostic model with regard to the remaining five predictors showed a similar discriminative ability of the R4U score in the validation set (AUC 0.61 (95% CI 0.56–0.66) compared to the development set. The updated prognostic model improved calibration (Fig. 1). Sensitivity increased from 11 to 23%.

## Discussion

We present an updated R4U-scorecard that is applicable in the first trimester of pregnancy to estimate the risk of adverse perinatal health outcomes, based on a comprehensive set of medical, obstetric, and non-medical risk factors (supplementary Fig. 1). By using a large external dataset and by applying a stepwise statistical approach to update the prognostic model and perform cross-validation, we have provided a comprehensive evaluation of this diagnostic tool^12–14^.

Our large multicentre prospective cohort included both low- and high-risk pregnancies derived from a population in which the model is aimed to be used. We applied domain validation. This is considered to be the broadest form of validation, leading to the strongest evidence that the prediction model can be generalised to new patients over time. The generalisability was underlined by the predictive value of the model in the validation set. A scorecard that is generalizable to new patients makes the subsequent institution of preventive strategies more relevant. We present a detailed description of the methodology used to update the prognostic model in several distinct steps. Validation studies of antenatal risk surveillance tools that include non-medical risk factors, such as a person’s socioeconomic status, are to our knowledge non-existent. The steps we present could be considered as a framework, and can be applied in other fields of study based on the elaborate description provided.

There are also several limitations that merit discussion. First, predictors are interconnected making it difficult to establish their independent contribution. For example, having a low household income might induce changes in one or more other risk factors such as housing conditions, but risk factors such as chronic diseases may also reduce labour supply and earnings^15–17^. In view of these complex relationships, our estimates and the resulting cumulative score, which assumes unidirectional causal associations, should be interpreted with caution.

Second, the development and validation of the models originated from a prospective cohort in The Netherlands, potentially limiting the generalisability outside the Dutch antenatal health care system. Additionally, the previously reported degree of selection bias in the C-RCT^7^, also applies to the results presented. A generally healthy population was included with a lower incidence of adverse pregnancy outcomes than the Dutch national average. Importantly, this bias is likely to cause underestimation of the discriminating power of the model.

Thirdly, we made some simplifications for easy clinical application of the R4U-scorecard. For example, all predictors and the outcome were dichotomised.

Both calibration and discrimination are useful aspects of a prediction model. However, in general discrimination is insensitive to errors in calibration, and considers the situation of classification in a pair of participants with and without the endpoint^18^.

By applying the stepwise statistical approach in order to update the predictors in the scorecard we primarily intended to improve calibration.

To further improve clinical decision making with the updated scorecard, a range of thresholds for high and low-risk participants could be considered to optimise the discriminative value. It is usually difficult to define an optimum threshold as empirical evidence for the relative weights of benefits and harms is often lacking. In our example considerations should weigh the potential of early identification of pregnant women at risk and the possibility to introduce preventive strategies early in the first trimester of pregnancy, against the potential harms of 'over-treatment'.

Moreover, to create a valuable decision tool for antenatal risk surveillance and preventive strategies, a prognostic model alone is not sufficient. Consecutive preventive strategies (e.g. care-pathways) prioritised at addressing risk factors with a high relative risk for adverse health outcomes together with comprehensive guidelines for preventive strategies for individual risks, need to be available and updated regularly to fit changes in daily clinical practices. Also, updating of the R4U prognostic scoring system may be needed to meet the local population.

Implementation of accurate prognostic models early in pregnancy provides room for preventive strategies and embodies potential to change daily practices and reduce early adversity in health outcomes. By updating the R4U-scorecard we have amended a clinical tool to guide these actions. Furthermore, we presented a framework for updating of a prognostic model with new information while keeping the prior information. This framework is relevant for wider implementation of prognostic models in clinical practice.

## Methods

Using external data from a national Cluster-Randomised Controlled Trial (C-RCT)^7^, we performed cross-validation of the R4U-scorecard with re-consideration of the additional effect of all predictors included in the scorecard. We then derived an updated version of the R4U-scorecard.

### Derivation cohort the healthy pregnancy 4 All-1 programme

The national HP4All-1 programme was conducted in The Netherlands from 2011 through 2014^9^. Two sub-studies within the programme combined public health and epidemiologic research. The first evaluated the effectiveness of programmatic preconception care, and the second evaluated the effectiveness of antenatal risk assessment with consecutive risk-guided care throughout pregnancy^8,19^.

### The antenatal risk assessment sub-study

The antenatal risk assessment sub-study was conducted as a C-RCT aiming to reduce adverse pregnancy outcomes by implementing a complex intervention^7^. The complex intervention consisted of three parts; (1) a first trimester risk surveillance using the R4U-scorecard, assessing both medical and non-medical risk factors known to be associated with adverse perinatal health outcomes (supplementary Fig. 1); (2) subsequent application of risk-specific care pathways; and (3) multidisciplinary consultation between care professionals from different echelons to discuss high-risk cases (e.g. health care organisations, public health care organisations, the office for legal or financial support).

Randomisation in this study took place at the level of the clusters, consisting of community midwifery practices or obstetric departments in hospitals. In the intervention arm, identification of specific risk factors implied a follow-up action such as tailoring care using risk-specific care pathways. In the control clusters, conventional obstetric care was provided. This consisted of screening by means of the ‘list of obstetric indications’ (LOI), which focuses on identification of single, manifest obstetric and medical risks, combined with individual care according to local protocols of obstetric care givers^6^.

The data from this C-RCT was used as external data to update the R4U-scorecard that was originally piloted in several hospitals and midwifery practices in Rotterdam from 2010 until 2011^20^.

### The R4U-scorecard

The primary basis for the R4U-scorecard was a simple scoring system in which all components had been selected and scores assigned both subjectively by expert consensus and objectively using available scientific literature, as described previously^10^.

Seventy-nine medical and non-medical dichotomised variables were incorporated in the R4U-scorecard, of which 76 pertain to the first trimester (supplementary Fig. 1). Key examples of non-medical risk factors include: low socioeconomic status, living in a deprived neighbourhood, ineffective social integration into society, and smoking.

Two types of variables were included in the first trimester risk surveillance: predictors and awareness items^7^. The first type of factor was incorporated in the R4U-scorecard as predictive factor and will be referred to as ‘predictors’ (50 items). The original weighing of each predictor was based on the relative risk for adverse pregnancy outcomes (e.g. babies born preterm and/or SGA). The scores of the individual items ranged from 0 to 3 points and these were added up to form a cumulative score (range 0–98 points). The cumulative score of the R4U-scorecard was developed using a simple approach assuming that all features are conditionally independent of each other given the class, based on Bayes’ rule^21^. The initial cut-off score was based on data from a pilot study; a score of 16 points or higher was selected to identify women in the upper 20% of risk scores^8,22^. A score above this cut-off implied a follow-up action via a multidisciplinary consultation between involved care professionals guided by a particular single, or a set of multiple, risk factors^7^.

Awareness items were incorporated to increase awareness for factors that could mediate the association between risk factors and adverse pregnancy outcomes, or to factors that are considered to be ‘red flags’ (26 items)^10,22^. All awareness items are indications for additional consideration or evaluation, and these items do not have a score. Examples of potential mediators are: ‘irredeemable financial debts’, and ‘previous referral to youth social services’, and an example of a red flag is ‘having no health care insurance’.

### Participants

Participants in the intervention arm of the HP4All-1 risk screening C-RCT were included in the current study if the following data was available; (1) a completed R4U-scorecard and (2) pregnancy outcome data collected in the follow-up period.

### Step 1. Data management and dealing with missing values

The primary outcome measure in the C-RCT was neonatal morbidity, defined as the combination of preterm birth (i.e. a delivery before 37 completed weeks of gestation), and/or having a SGA baby (i.e. a birth weight below the 10th centile adjusted for parity, gestational age, and gender, based on the Dutch reference curves)^23^. We compared maternal, pregnancy, and prior-pregnancy predictors in uncomplicated pregnancies with pregnancies followed by perinatal morbidity (Table 1).

Seven percent of the participants had at least one missing value within the predictor items, and complete case analysis would have reduced the total sample by 19%. A multiple imputation approach was therefore used to account for missing values in predictors^24^. Predictor variables and outcome variables were included to inform the process, forming 20 datasets using multiple imputations with chained equations^25^. Fifteen predictors with a low incidence were excluded from the multiple imputation process since this might have resulted in computational instability and unreliable estimates. We defined a low incidence as an incidence below 2% of the total sample size. The imputed data was then used to update the original prognostic model (step 3).

### Step 2: Cross-validation of the original prognostic model

Cross-validation was based on the inclusion date of participants within the HP4All-1 programme^11^. Participants before September 2014 were included in the development set and participants from September onward were included in the validation set. This date was chosen based on a second training session provided to all health care providers that implemented the R4U-scorecard in routine practice. Domain validation was performed first on complete cases to test the generalisability of the prognostic model across different domains, including participants from different health care settings (i.e. community midwifery practices, secondary and tertiary hospital care)^14^. Validation was assessed with calibration plots and by computing the area under the receiver operating characteristic curve (AUC) with a 95% confidence interval (CI)^26,27^. Calibration was defined as the agreement between the probabilities of neonatal morbidity, as predicted by the prognostic model, and the observed frequencies. Discrimination was defined as the ability of the original model to distinguish between women who will have a preterm and/or SGA baby and those who will not. Sensitivity and specificity were calculated at the pre-specified cut-off R4U score of 16 points.

### Step 3: Updating of the original prognostic model

The process of updating the original prognostic model consisted of four steps. The multiple imputed data was used to re-estimate the effect of each predictor in the model for updating^13^. The development set was used to update the prognostic model. The validation set was used to test generalisability.

In the first step we determined which predictors were to be re-estimated by assessing their additional predictive value on top of the cumulative R4U-score. Predictors that were assessed separately in the second trimester of pregnancy were not evaluated (three items) and predictors that related to prior pregnancy characteristics were evaluated in multiparous women only (five items). A reference model was based on a univariate logistic regression model describing the association of the cumulative R4U-score with perinatal morbidity. Separate bivariate logistic regression models were constructed adding single predictors one at a time. Each nested, bivariate logistic regression model was tested separately against the reference model. Predictors were categorised as ‘candidate predictors’ if the p value of their association with adverse pregnancy outcomes independent of the total R4U score was below 0.20, with reference to the Wald test. Final selection of all candidate variables for the fully updated model was based on backward elimination of variables with a p value above 0.20.

In the second step a heuristic shrinkage factor was added to adjust β-coefficients of all included predictors for overfitting and to avoid extreme predictions when applied to new participants^13,28,29^.

The shrinkage factor was estimated as follows^29^:

$$ \frac{{{\text{Model }}\chi^{{2 }} {-}\left( {{\text{degrees of freedom }} - \left. 1 \right)} \right.}}{{{\text{Model }}\chi^{{2 }} }} $$

The number of degrees of freedom in this case is the total number of degrees of freedom that is considered in the process of selecting from all predictors, plus all covariates fitted in the model.

The third step consisted of an evaluation of the obtained multivariable model by exploring the β-coefficients and their corresponding sign and size. Because all predictors were initially incorporated in the R4U-scorecard based on their *positive* association with adverse pregnancy outcomes, a *negative* sign of the β-coefficient in the current multivariable model was considered counterintuitive. Counterintuitive signs observed in multivariable models can be explained by correlations between predictors and therefore careful evaluation of the model obtained is necessary^29^. External information from recent literature and expert opinion was sought if a sign was counterintuitive in both univariate and multivariable analyses to finalise the model selection.

In the fourth and final step, we determined the additional effect of each predictor. Hereto we divided the β-coefficients obtained from the fully updated model, by the value of the coefficient corresponding with one point increase in the cumulative R4U-score, after shrinkage and evaluation of the sign had been accounted for.

### Step 4: Assessing generalisability in the validation set using the updated model

To assess the predictive value of the updated model we used the validation set. Validation was assessed with calibration plots and by computing the area under the receiver operating characteristic curve. Sensitivity and specificity of the original and update score were compared in the validation set.

### Transparency declaration

The lead author affirms that this manuscript is an honest, accurate, and transparent account of the study being reported; that no important aspects of the study have been omitted; and that any discrepancies from the study as planned (and, if relevant, registered) have been explained.

### Ethical consideration

The study was reviewed by the Medical Ethical Review Board of the Erasmus MC. All research was performed in accordance with the relevant regulations. The Board provided a waiver for the need to obtain consent at the individual level according to Dutch law as all procedures were essentially accepted care, and data were analysed anonymously (MEC-2012-322).

## Supplementary information

Supplementary file1 (PDF 67 kb)

## Data Availability

The datasets generated during and/or analysed during the current study are available from the corresponding author on request.
