# Supplementary material for: Validation of a prognostic model for adverse perinatal health outcomes
Source: Sci Rep. 2020 Jul 9;10:11243. doi: 10.1038/s41598-020-68101-3 (PMC7347528; doi:10.1038/s41598-020-68101-3)
Supplement: Supplementary file 1 — Supplementary file1 (PDF 67 kb) [file 41598_2020_68101_MOESM1_ESM.pdf]

SUPPLEMENTARY FILES

Title

Validation of a prognostic model for adverse perinatal health outcomes

Authors

Jacqueline Lagendijk[1], Ewout W. Steyerberg[2 3], Leonie A. Daalderop[1], Jasper V. Been[1,2,4], Eric A.P. Steegers[1], Anke G. Posthumus[1]

Supplementary figure 1: The R4U-scorecard

**Name**		**Study ID**			**G: P:**	**Date of Birth**	
**Practice**		**Zip Code**			**Date of booking visit**	**Due date**	
**SOCIAL**			**YES**	**NO**	**LIFESTYLE (continuation)**	**YES**	**NO**
**Social situation**					**Nutrition**		
**Single mother**			1 **(6)**	0	Vegetarian, vegan or macrobiotic diet		
Relationship problems > 3 months					No daily vegetable intake		
**Experience of inadequate social support**			1 **(7)**	0	No daily fruit intake		
Domestic violence			2	0	**Body weight**		
Previous referral to children's social services					BMI < 18	1	0
**Work and income**					BMI 30 - 35		
Unemployed (> 3 months)			1	0	BMI > 35	2	0
Standing labour			1	0	**GENERAL HISTORY**		
Working hours > 32 and stressful			2	0	**Disorders**		
**Netto family income < 1000 euro**			2 **(7)**	0	Chronic maternal illness (as described in script)	2	0
Irredeemable Financial debts					Annual consultation GP or physician		
Partner unemployed			1	0	Hemoglobinopathy	2	0
**Education**					Refuses blood transfusion (Jehovah's Witness)		
Low education level (< 7 years) or illiterate			2	0	**Medication**		
**Neighbourhood**					Prescribed medication	2	0
Housing problems					Over-the-counter drugs	2	0
Deprived neighbourhood*			2	0	No preconceptional folic acid use	2	0
**ETHNICITY**					**Infectious diseases**		
**Ethnicity**					(Treated for) sexually transmitted disease last year	2	0
Surinamese - Hindo			2	0	Promiscuity	2	0
Surinamese - Creole			2	0	At risk for Toxoplasmosis	1	0
Surinamese - Javanese			2	0	At risk for Rubella	1	0
Antillean / Aruban			2	0	**Psychiatry**		
Cape Verdian			2	0	History of psychiatric admission / positive family history (1st degree)	3	0
Turkish			2	0	Current use of psychiatric medication		
Maroccan			2	0	Current psychiatric problems	3	0
Non-Western other			2	0	**OBSTETRIC HISTORY**		
**Language / communication**					**History**		
Language barrier (limited Dutch or English)					Nulliparous	2	0
Exclusively communication by interpreter					Recurrent miscarriage (2 or more)	1	0
Mentally disabled					Interpregnancy interval < 6 months	3	0
**REPRODUCTIVE FACTORS**					**Preterm birth (< 37 weeks)**	3 **(8)**	0
**General**					Low Apgarscore < 7 after 5 minutes	3	0
Uninsured					**Small for gestational age (SGA) baby (<p10)**	3 **(16)**	0
**Family planning / age**					Previous child with major congenital anomalies	3	0
Unwanted pregnancy			1	0	Stillbirth (22 weeks - 7 days postpartum)	3	0
Unplanned, but wanted pregnancy					Shoulder dystocia		
Assisted reproduction (ICSI/IVF/IUI/oocyte donation)			2	0	Instrumental delivery		
Teenage pregnancy (≤ 18 yeas)			3	0	Caeserean section		
Advanced maternal age (≥ 40 jaar)			2	0	Gestational diabetes	1	0
**Obstretical**					Manual placental removal / postpartum haemorrhage		
Start antenatal care 12 - 14 weeks					Placental abruption	3	0
Late start antenatal care > 14 weeks			3	0	(Pre)eclampsia or HELLP syndrome	3	0
**LIFESTYLE**					**Family**		
**Intoxication**					Major congenital anomaly in first degree relative	1	0
Preconceptional smoking (past 6 months)			1	0	Other (as described obstetric indication list)		
Smoking during first trimester			2	0	**Result booking bloods**		
Smoking during second trimester			2	0	Irregular antibodies		
Preconceptional alcohol use (past 6 months)					Hepatitis B	2	0
Alcohol use during first trimester			1	0	HIV	2	0
Alcohol use during second trimester			1	0	Lues	2	0
Preconceptional illicit drug use (past 6 months)			1	0	**RESULT**	**Score**	
Illicit drug use during first trimester			3	0	Social		
Illicit drug use during second trimester			3	0	Ethnicity		
					Care		
					Life style		
					General history		
					Obstetrical history		
					Lab results booking bloods		
						**Cumulative**	

All items in the R4U-scorecard with scores for predictor items. Predictor items that were adjusted in the updated version are marked with the score from the original R4U and the updated score in bold brackets.
